# Caloric Restriction Is More Efficient than Physical Exercise to Protect from Cisplatin Nephrotoxicity via PPAR-Alpha Activation

**DOI:** 10.3389/fphys.2017.00116

**Published:** 2017-03-02

**Authors:** Gabriel R. Estrela, Frederick Wasinski, Rogério O. Batista, Meire I. Hiyane, Raphael J. F. Felizardo, Flavia Cunha, Danilo C. de Almeida, Denise M. A. C. Malheiros, Niels O. S. Câmara, Carlos C. Barros, Michael Bader, Ronaldo C. Araujo

**Affiliations:** ^1^Departamento de Biofísica, Universidade Federal de São PauloSão Paulo, Brazil; ^2^Departamento de Medicina, Disciplina de Nefrologia, Universidade Federal de São PauloSão Paulo, Brazil; ^3^Departamento de Imunologia, Instituto de Ciências Biomédicas, Universidade de São PauloSão Paulo, Brazil; ^4^Departamento de Clinica Médica, Universidade de São PauloSão Paulo, Brazil; ^5^Departamento de Nutrição, Escola de Nutrição, Universidade Federal de PelotasPelotas, Brazil; ^6^Max-Delbruck Center for Molecular MedicineBerlin, Germany

**Keywords:** cisplatin nephrotoxicity, inflammation, caloric restriction, exercise, PPAR-alpha

## Abstract

The antineoplastic drug cisplatin promotes renal injury, which limits its use. Protocols that reduce renal cisplatin toxicity will allow higher doses to be used in cisplatin treatment. Here, we compare physical exercise and caloric restriction (CR) as protocols to reduce cisplatin renal injury in mice. Male C57BL/6 were divided into four groups: Control, cisplatin, exercise + cisplatin, and 30% CR + cisplatin. Animals were injected with a single dose of cisplatin (20 mg/kg i.p.) and sacrificed 96 h after injection. Quantitative real time PCR, histological analyses, immunohistochemistry, and biochemical measurements were performed to investigate renal injury, necrosis, apoptosis, and inflammatory mechanisms. Both protocols protected against cisplatin renal injury, but CR was more effective in reducing uraemia and renal necrosis. The CR + Cisplatin group exhibited reduced serum IL-1β and TNF-α levels. No differences were noted in the renal mRNA expression of cytokines. Both interventions reduced apoptosis, but only the CR + Cisplatin group decreased TNFR2 protein expression. PPAR-α was activated in mice after CR. An antagonist of PPAR-α blocked the protective effect of CR. Both interventions attenuated the nephrotoxicity caused by cisplatin injection, but CR + Cisplatin showed a better response by modulating TNFR2. Moreover, part of the CR benefit depends on PPAR-α activation.

## Introduction

Cisplatin is an antineoplastic drug used to treat multiple cancers, including those of the head, neck, lungs, testicles, breast, and ovaries. Cisplatin may induce ototoxicity, gastrotoxicity, hepatotoxicity, neurotoxicity, myelosuppression, and allergic reactions, but the main side effect is nephrotoxicity. Between 20 and 30% of the patients treated with cisplatin experience acute kidney injury (Miller et al., 2010). The mechanisms of cisplatin nephrotoxicity involve oxidative stress, apoptosis, inflammation, and fibrinogenesis. In addition, high cisplatin concentrations induce proximal tubule cell necrosis (Lieberthal et al., 1996).

Tumor necrosis factor receptor 2 (TNFR2) is important in cisplatin-induced acute kidney injury. Knockout mice for this receptor exhibit attenuated side effects of cisplatin nephrotoxicity, including diminished acute tubular necrosis, lowered pro-inflammatory cytokine expression, and diminished leukocyte infiltration and apoptosis (Ramesh and Reeves, 2003).

Physical exercise can alter the immune system, affecting innate immune response cells, such as neutrophils, macrophages and NK cells, the humoral system (antibodies and cytokines), and acute phase proteins (Nieman and Nehlsen-Cannarella, 1994; Pedersen et al., 2003; Wasinski et al., 2013).

Recently, physical exercise was shown to diminish cisplatin-induced acute kidney injury by promoting interleukin-6 and heme oxygenase-1 expression in the kidney, decreasing inflammation and cell death (Miyagi et al., 2014).

Caloric restriction (CR) includes a wide variety of interventions that result in nutrient reduction and energy consumption without causing desnutrition. CR augments life expectancy and resistance to multiple forms of acute stress by controlling inflammation, oxidative stress and apoptosis (Sinclair, 2005; Brown-Borg, 2006; Bishop and Guarente, 2007). Moreover, CR reduces the damage caused by ischaemia and reperfusion in the heart, brain and kidney (Yu and Mattson, 1999; Chandrasekar et al., 2001; Ahmet et al., 2005; Mitchell et al., 2010), ameliorates insulin sensitivity, augments antioxidant proteins and diminishes inflammatory markers such insulin grown factor-1 signaling (Mitchell et al., 2010).

Knowing that inflammation is a key factor in cisplatin nephrotoxicity and that CR and exercise are two non-pharmacological interventions to attenuate inflammation, we decided to compare the roles of both interventions in cisplatin-induced acute kidney injury.

## Materials and methods

### Animals

**Figure 1:**
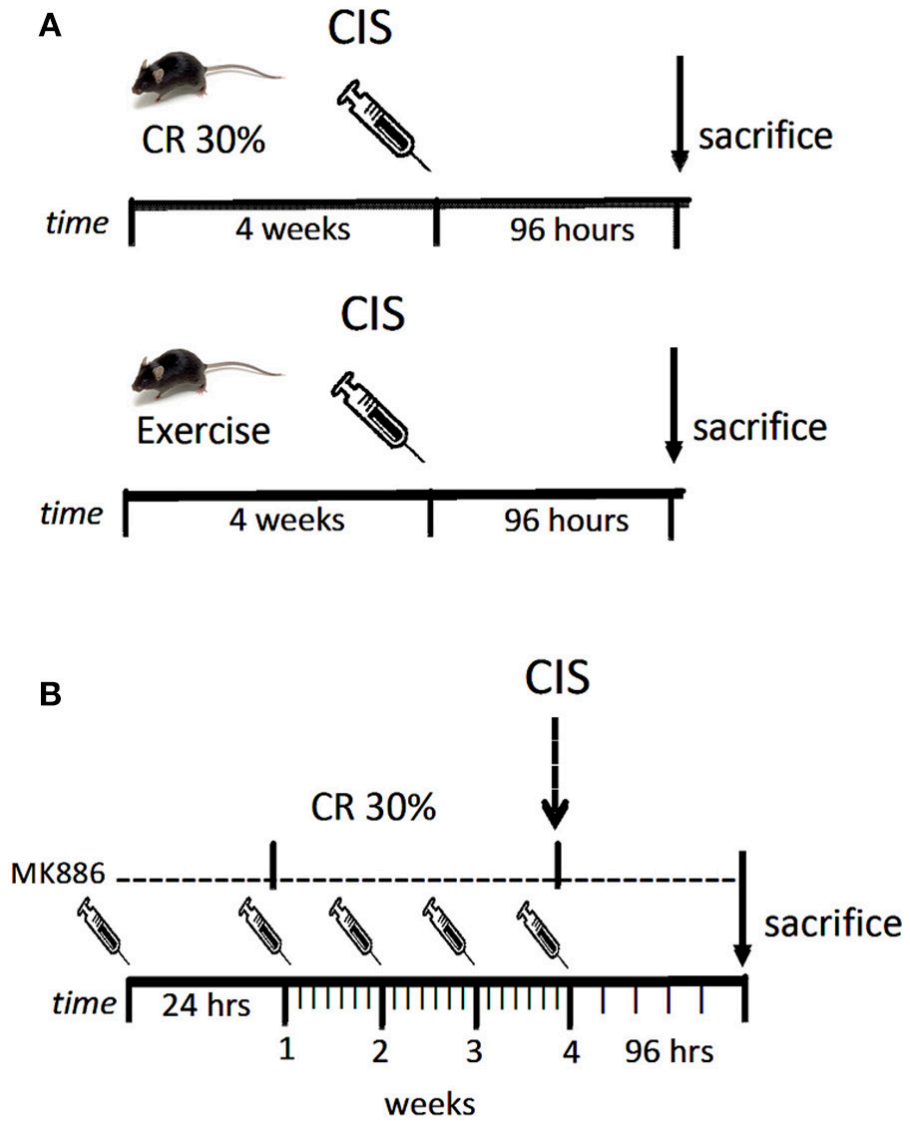
**Drug treatment scheme**. Mice were submitted to exercise or caloric restriction for 4 weeks prior to cisplatin injection **(A)**. Mice were treated with MK886 24 h prior to initiate caloric restriction. Treatment with MK886 were kept into the day of sacrifice. Cisplatin was administrated in the end of the 4th week of caloric restriction and mice were sacrificed 96 h after the injection **(B)**. *n* = 6 per group.

Male C57BL/6 mice weighing 25–30 g and of 12 weeks of age were used for these experiments. Single doses of cisplatin (20 mg/kg; Bergamo, Taboão da Serra, Brazil) were injected intraperitoneally (i.p.), and the animals were sacrificed 96 h after the injection. The animals were obtained from the Animal Care Facility at the Federal University of São Paulo (UNIFESP). All animals were housed individually in standard cages and had free access to water and food. All procedures were previously reviewed and approved by the internal ethical committee of the institution (Figure 1).

### Experimental design

Mice (*n* = 6 per group) were divided in four different groups: (1) control mice (Control), (2) cisplatin-treated mice (CIS), (3) exercise + cisplatin-treated mice (EX + CIS), and (4) 30% CR + cisplatin-treated mice (CR + CIS).

### CR protocol

The amount of food eaten *ad libitum* was determined by weighing the remaining food on a daily basis for 1 week. CR was applied for 4 weeks by feeding mice 70% of this amount on a daily basis.

### Exercise protocol

The animals were subjected to swimming sessions in a swimming system adapted for mice with water heated to 30°C. The swimming sessions began with 15 min in the first week and gradually increased in length until the mice were able to swim for 60 min a day. After the adaptation period (2 weeks), mice were subjected to 5 swimming sessions per week for 4 weeks.

### Renal function

Serum creatinine and urea levels were used to determine renal function. Blood was collected by heart puncture. All samples were analyzed with colorimetric assays, using commercial kits to detect creatinine and urea (both from Labtest, Lagoa Santa, Brazil).

### Quantification of gene expression

Kidney samples were frozen at −80°C. Total RNA was isolated using TRIzol reagent according to the manufacturer's instructions (Invitrogen, Carlsbad, CA). First-strand cDNA was synthesized using the High Capacity cDNA Reverse Transcription kit (Applied Biosystems). Real-time PCR was performed using two systems: The TaqMan system (Applied Biosystems, Carlsbad, CA) using probes for IL-6 (mm00446190-m1), TNF-α (mm00443258-m1), and GAPDH (mm99999915-g1), and the SYBR Green assay (Thermo Scientific, Waltham, MA) using the primers described in Table 1. The cycling conditions for both the TaqMan and SYBR Green primers were as follows: 10 min at 95°C, followed by 45 cycles of 30 s at 95°C, 30 s at 60°C, and 30 s at 72°C. Standard curves were performed for each primer pair to check the amplification efficiency. Target mRNA expression was normalized to β-actin for SYBR and to GAPDH for TaqMan and expressed as a relative value using the comparative threshold cycle (Ct) method (2^−ΔΔCt^) according to the manufacturer's instructions. The expression levels of the genes of interest were normalized to the control group. All samples were run in duplicates.

**Table 1:** **Base pair sequences of primers used in real-time PCR assays**.

**Gene**	**Primers**
β-actin	5′-CTGGCCTCACTGTCCACCTT-3′
	5′-CGGACTCATCGTACTCCTGCTT-3′
IL-1β	5′-AGGAGAACCAAGCAACGACA-3′
	5′-CGTTTTTCCATCTTCTTCTTTG-3′
BAX	5′-CGGCGAATTGGAGATGAACTG-3′
	5′-GCAAAGTAGAAGAGGGCAACC-3′
BCL-2	5′-ACCGTCGTGACTTCGCAGAG-3′
	5′-GGTGTGCAGATGCCGGTTCA-3′
TNFR1	5′-TACATCCATCAGGGGTCACT-3′
	5′-AGGCACAACTTCATACACTC-3′
TNFR2	5′-GTCGCGCTGGTCTTCGAACTG-3′
	5′-GGTATACATGCTTGCCTCACAGTC-3′
CPT-1A	5′-GTTCCCCGCGAGTCCCTCCA-3′
	5′-GCTTGACATGCGGCCAGTGGT-3′
CPT-2	5′-CCAAGCACAGTGTGGGCGAGC-3′
	5′-GGTCAAAGCCCTGGCCCATCG-3′
CYP4A	5′-GGAGGATGCTAACCCCAGCCTTCC-3′
	5′-GCCAGCCGTTCCCATTTGTCTAGC-3′
PPAR-α	5′-ATGCCAGTACTGCCGTTTTC-3′
	5′-TTGCCCAGAGATTTGAGGTC-3′

*IL-1β, interleukin 1β; BAX, Bcl-2-associated X; BCL-2, B-cell lymphoma 2; TNFR1, tumor necrosis factor receptor 1. TNFR2, tumor necrosis factor receptor 2; CPT-1A, carnitine palmitoyltransferase 1A; CPT-2, carnitine palmitoyltransferase 2; CYP4A, cytochrome P450 4A; PPAR-α, peroxisome proliferator-activated receptor alpha*.

### Histological analyses

Formaldehyde-fixed paraffin sections of kidneys were stained with haematoxylin and eosin (H&E). Optic light microscopy was employed to analyse the samples. Images were acquired at × 40 magnification. Epithelial desquamation, cellular debris, flattening of the epithelium, presence of cylinders, and dilation of the tubular lumen were used as criteria for tubular injury. The injuries were graded using a scoring procedure, in which I = 0–10% of the total kidney area was compromised, II = 11–25, III = 26–50, and IV ≥ 50%.

### Immunohistochemistry

Localization of cleaved caspase-3 was assessed in paraffin-embedded tissue sections. As described previously, the slides were deparaffinised and rehydrated. Antigens were retrieved in a citrate buffer solution (pH 6) at 95°C. The endogenous peroxidase activity was blocked with 3% hydrogen peroxide solution, and the sections were additionally blocked with Protein Block Solution (DAKO, Glostrup, Denmark). The slides were incubated with the primary antibody (diluted 1:1,000, Cell Signaling Technologies, Beverly, MA) or isotype non-specific IgG as a negative control, followed by incubation with the labeled EnVision polymer (DAKO, Glostrup, Denmark) using two sequential 30 min incubations at room temperature. The staining was developed by incubating for 1–3 min with 3,39-diaminobenzidine plus substrate chromogen, which stains the specific antigen brown. Haematoxylin counterstaining was also performed.

### Analysis of cytokines in serum

Serum samples were stored at −80°C. The panel used for the Bio-plex Mouse cytokine/chemokine assay (Bio-Rad, Hercules, CA) included the following cytokines: MCP-1, TNF-α, IL1-β, IL-6, and IL-10. Testing was conducted in accordance with the procedures previously described by the manufacturer. All the samples were run in duplicates.

### Enzyme-linked immunosorbent assay

Kidney samples were stored at −80°C. Protein was extracted with RIPA Buffer. Kidney TNF receptor levels were quantified using Quantikine ELISA mouse TNFR1 and mouse TNFR2 (R&D Systems, Minneapolis, MN), respectively, according to the instructions provided by the manufacturer. All the samples were run in duplicates.

### Western blotting

For each kidney sample total protein was isolated using RIPA buffer (25 mM Tris-HCl pH 7.6, 150 mM NaCl, 1% NP-40, 1% sodium deoxycholate, 0.1% SDS) and global protein was measurement by BCA™ Protein Assay Kit (Pierce, EUA). Around 50 μg of tissue protein was utilized for SDS-PAGE electrophoresis on 12.5% polyacrylamide gels. The immunostaining was performed with primary antibodies (Anti-Cofilin/1:1,000, Abcam, USA, Anti-PPAR-α/1:300, Abcam, Anti-CPT1A/1:1,000, Abcam, Anti-CPT-2/1:500, Abcam and Anti-Beta Actin/1:5,000, Cell Signaling, USA), followed by conjugated secondary antibodies (anti-mouse or anti-rabbit peroxidase/1:250.000, Sigma, USA). After, the nitrocellulose membrane was revealed by chemiluminescence methods using the ECL kit (Millipore, USA), and the images were acquired on Amersham Imager 600 equipment (GE Healthcare, UK). The SyncMaster 740 n software device (GE Healthcare, UK) were used to analyze and quantify the gel bands. The experiment was reapeated twice.

### PPAR-alpha antagonist treatment

Male C57BL/6 mice weighing 25–30 g and 12 weeks of age were injected i.p. with 3 mg/kg of MK886 (Cayman Chemicals, Ann Arbor, MI) 1 day prior to beginning CR and received one dose daily until the day of sacrifice (Figure 1).

### Statistical analysis

All data are presented as the mean ± s.e.m. Different results between the groups were compared using analysis of variance (one-way ANOVA). The value for statistical significance was established at *P* < 0.05. All statistical analyses were performed using GraphPad Prism (GraphPad, La Jolla, CA), all the other comparisons without the presence of brackets were not significant.

## Results

### Exercise and CR ameliorate cisplatin renal injury

**Figure 2:**
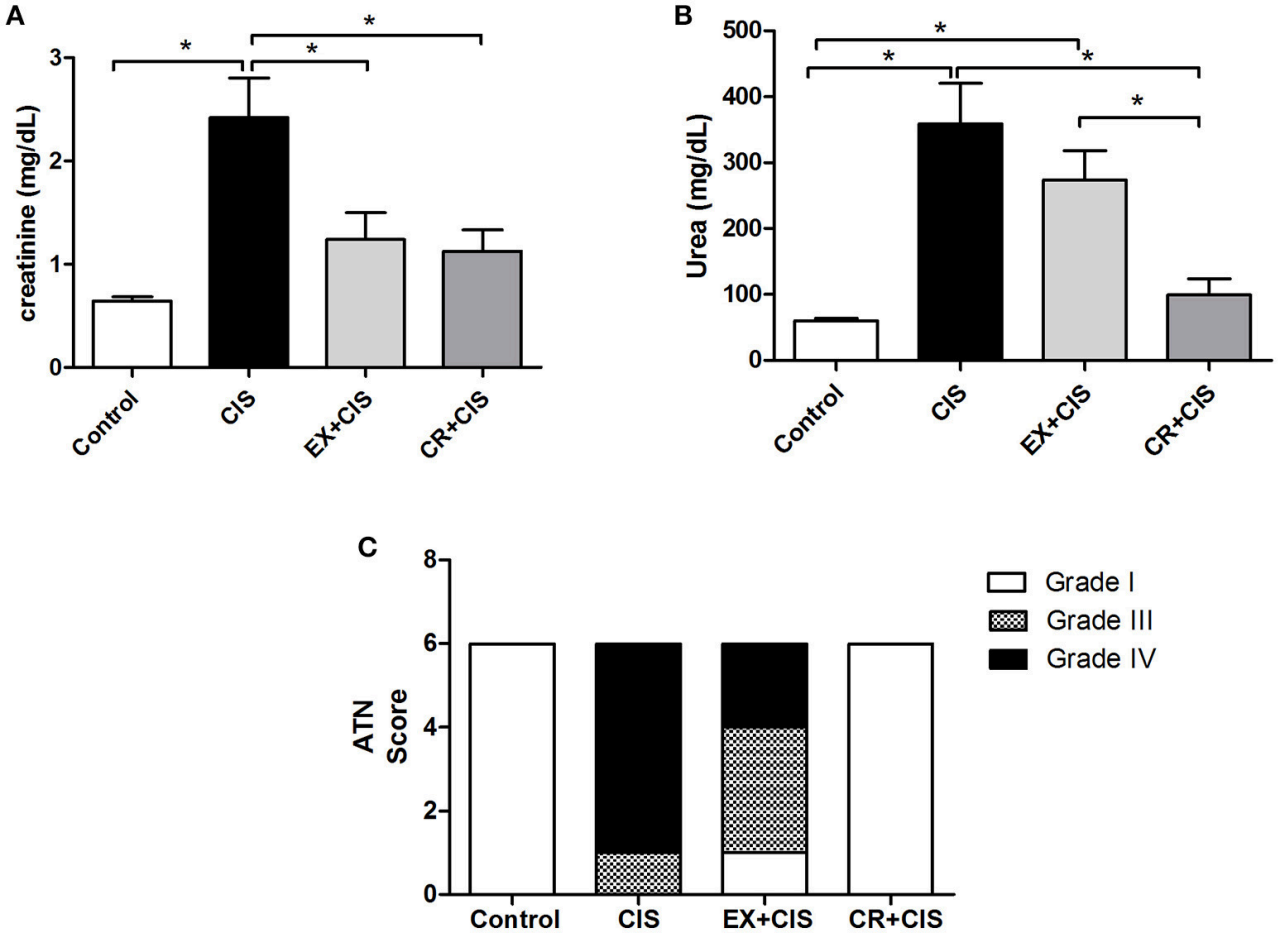
**Renal function and ATN score**. Serum creatinine is reduced in both the CR+CIS and EX+CIS groups compared with the animals that received only cisplatin **(A)**. Urea is reduced only in the CR+CIS group **(B)**. The CR+CIS group presented a better score in the ATN test, showing less necrosis in renal tissue. CIS, cisplatin; EX+CIS, exercise + cisplatin; CR+CIS, caloric restriction + cisplatin; ^*^p < 0.05. *n* = 6.

It was previously shown that 7 weeks of chronic exercise on a training treadmill protected mice from cisplatin-induced renal injury (Miyagi et al., 2014). Here, we show the effects from CR and swimming exercise. Figure 2A shows the reduced serum creatinine levels in both the CR+CIS and EX+CIS groups compared with the animals that received only cisplatin. However, the serum urea levels were only reduced in the CR+CIS group (Figure 2B). The histology analyses showed that CR completely protected the kidney from tubular necrosis induced by cisplatin, and exercise was not as effective (Figure 2C). These results suggest different mechanisms of protection for CR and exercise.

**Figure 3:**
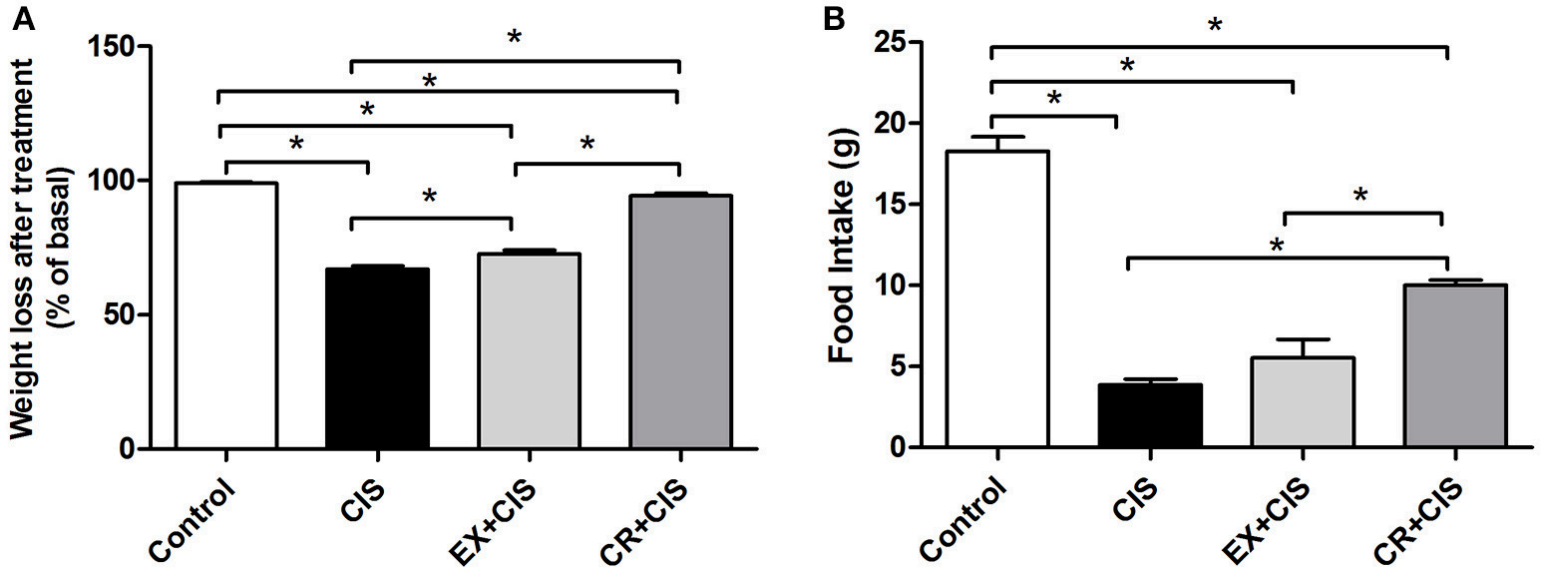
**Body weight and food intake. (A)** Changes in body weight and **(B)** food intake after cisplatin injection. CIS, cisplatin; EX+CIS, exercise + cisplatin; CR+CIS, caloric restriction + cisplatin. ^*^p < 0.05. *n* = 6.

Figure 3 shows the reduced effect on body weight loss and changes in food intake in both the CR+CIS and EX+CIS groups when compared to animals treated with only cisplatin.

### Serum IL-1β and TNF-α are reduced in the CR+CIS group

**Figure 4:**
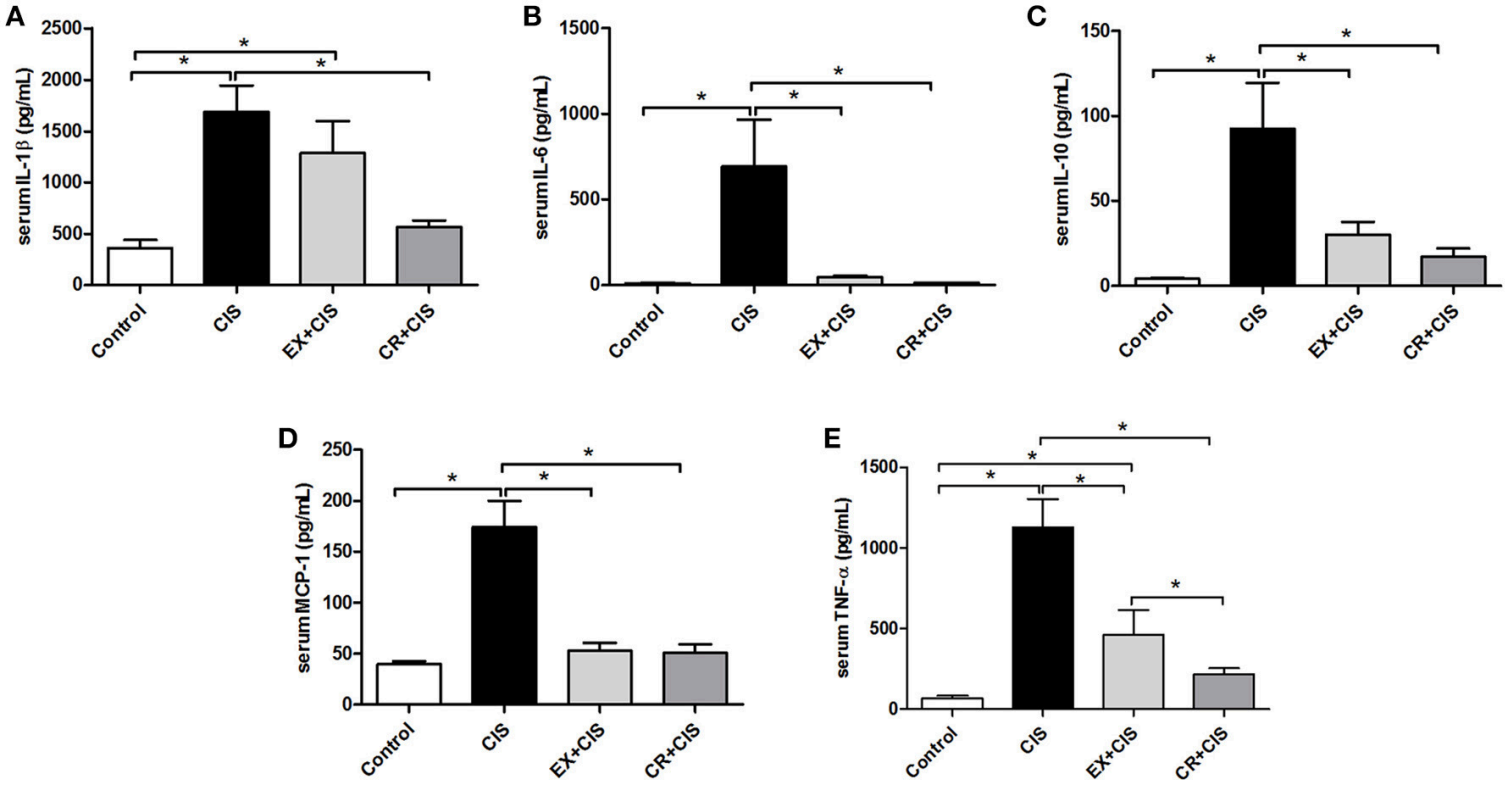
**Serum cytokines**. Serum leveols of **(A)** IL-1β, **(B)** IL-6, **(C)**, IL-10, **(D)** MCP-1, and **(E)** TNF-α. CIS, cisplatin; EX+CIS, exercise + cisplatin; CR+CIS, caloric restriction + cisplatin. ^*^p < 0.05. *n* = 5–6.

To identify the different mechanisms of kidney protection against cisplatin treatment for CR and exercise, we measured cytokine levels in mouse serum. The levels of IL-1β and TNF-α were lower in the CR+CIS group than in the EX+CIS group. We did not see significant differences between IL-6, MCP-1, and IL-10 expression in the CR+CIS and EX+CIS groups (Figure 4).

### The local cytokine formation is similar in kidneys from both treatment groups

**Figure 5:**
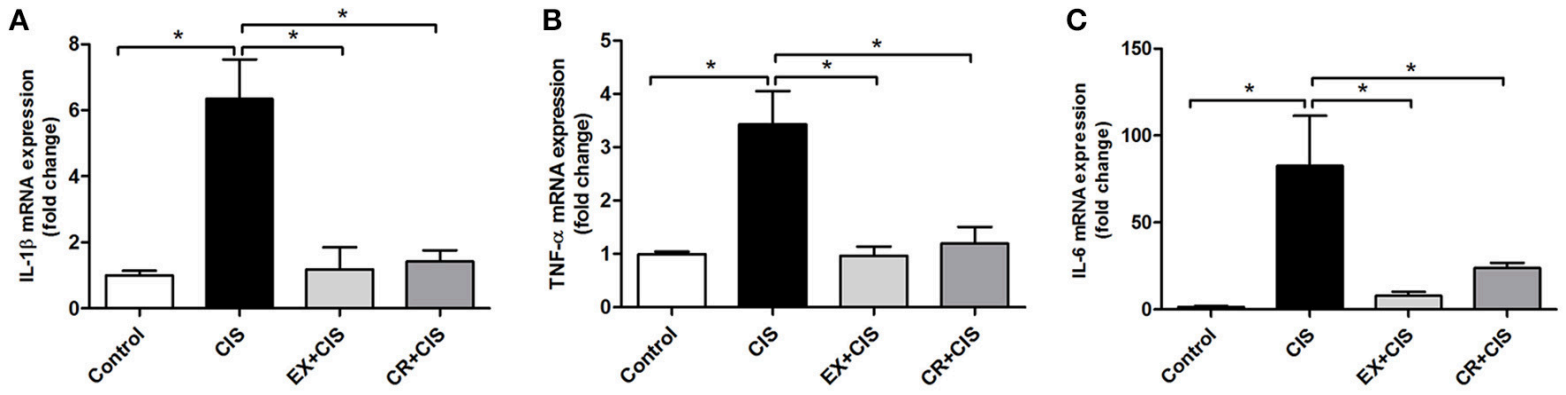
**Renal mRNA expression of cytokines**. The EX+CIS and CR+CIS groups exhibited reduced **(A)** IL-1β, **(B)** TNF-α, and **(C)** IL-6 mRNA expression in the kidney compared to the CIS group, but there were no local differences between the expression of these genes in the CR+CIS and EX+CIS group kidneys. CIS, cisplatin; EX+CIS, exercise + cisplatin; CR+CIS, caloric restriction + cisplatin. ^*^p < 0.05. *n* = 5–6.

The differences in IL-1β and TNF-α serum levels were not explained by local kidney production of these cytokines, at least at the mRNA expression level. Both the CR+CIS and EX+CIS groups exhibited similar mRNA expression of both cytokines (Figure 5).

### Both interventions reduced apoptosis, but only the CR+CIS group decreased TNFR2 protein expression

**Figure 6:**
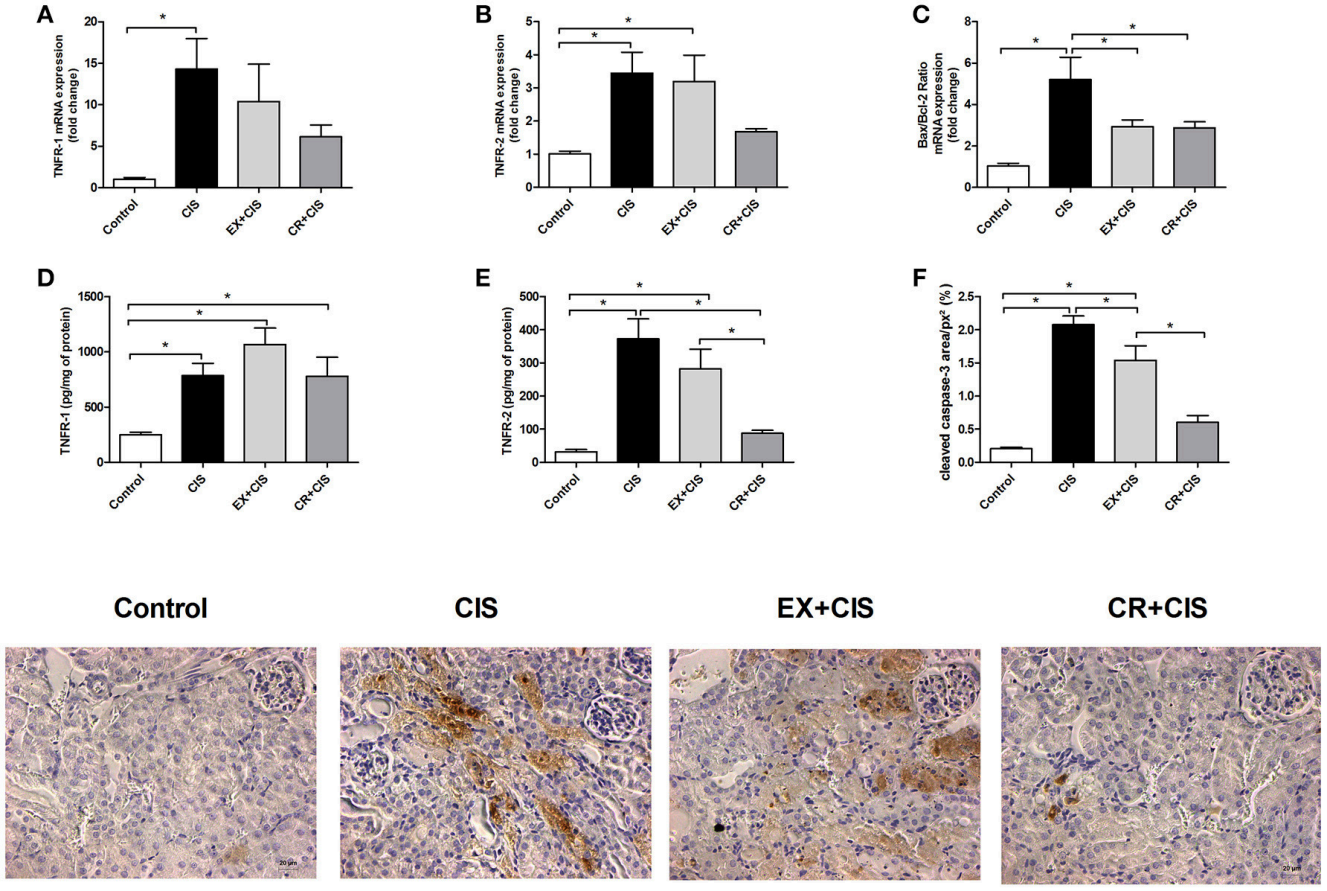
**Protein and mRNA expression of apoptotic factors**. Although the Bax/Bcl-2 ratio is similar in the CR+CIS and EX+CIS groups, TNFR2 protein expression was reduced in the CR+CIS group compared to the CIS and EX+CIS groups. **(A,B,D,E)** mRNA expression and renal protein assay of TNFR1 and TNFR2 by ELISA. **(C)** Bax/Bcl-2 ratio. **(F)** Cleaved caspase-3 present in kidney tissues. The section images illustrate the result in panel **(F)**. CIS, cisplatin; EX+CIS, exercise + cisplatin; CR+CIS, caloric restriction + cisplatin. ^*^ *p* < 0.05. *n* = 5–6.

Because the CR+CIS group exhibited reduced tubular necrosis compared to the EX+CIS group, we checked the expression of genes related to apoptosis (Figures 6A–C). The CR+CIS and EX+CIS groups had similar apoptosis activation, as shown by the similar Bax/Bcl-2 ratios in Figure 6C. On the other hand, TNFR2 protein expression was reduced in the CR+CIS group compared to the EX+CIS group (Figure 6E). Cleaved caspase-3, one of the main effectors of apoptosis, was also reduced in the CR+CIS group compared with the EX+CIS group, as analyzed by immunohistochemistry (Figure 6F). Together with the reduced necrosis shown in Figure 2C, these results suggest a different mechanism for CR protection against cisplatin renal injury. No significant difference where observed in TNFR-1 (Figure 6D).

### CR up-regulates mRNA expression of PPAR-alpha and its targets genes in kidneys

**Figure 7:**
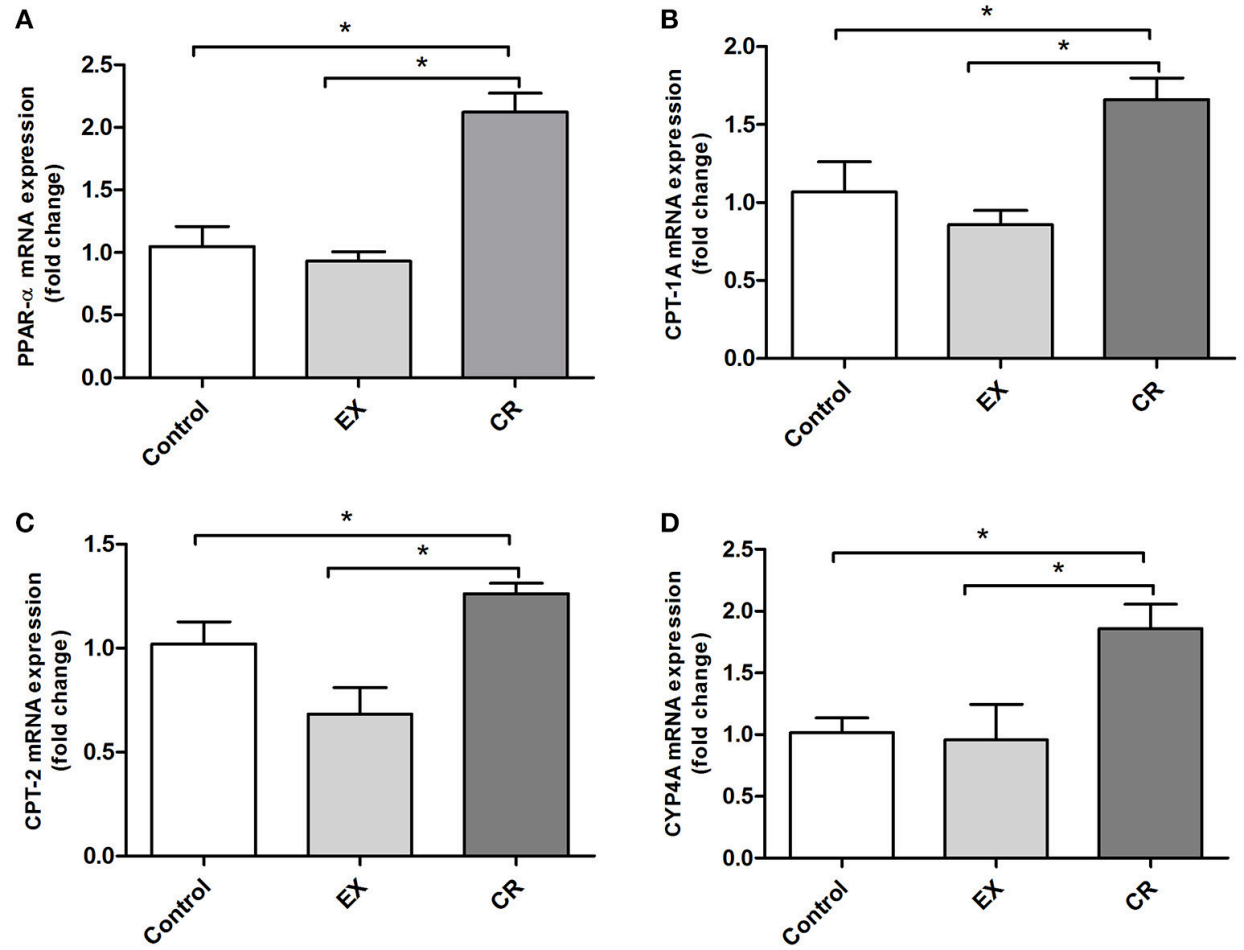
**PPAR-alpha and its target genes profile expression in the kidney**. CPTs and CYP4A are targets of PPAR-alpha activation, CPTs acts in Beta oxidation of long chain fatty acids while CYP4A participates in omega hydroxilation of medium chain fatty acids. Renal mRNA expression of **(A)** PPAR-alpha, **(B)** CPT-1A, **(C)** CPT-2, and **(D)** CYP4A in the kidneys of animals without cisplatin injection. CR activates PPAR-a target genes. EX, physical exercise; CR, caloric restriction. ^*^p < 0.05. *n* = 5–6.

We measured the mRNA expression of PPAR-α and its target genes in animals that underwent exercise and CR without cisplatin injection. The mRNA expression of PPAR-α and its targets genes were increased in the CR group (Figures 7A–D). PPAR-α activates lipid oxidation via the activation of acyl-CoA oxidase, the first enzyme in the beta-oxidation pathway. The activation of PPAR-α may be involved in the mechanism underlying the better renal protection against cisplatin injury observed in mice treated previously with CR.

### CR and exercise increases PPAR-alpha expression but only CR up-regulates CPT-1A and CPT-2 in renal tissue

**Figure 8:**
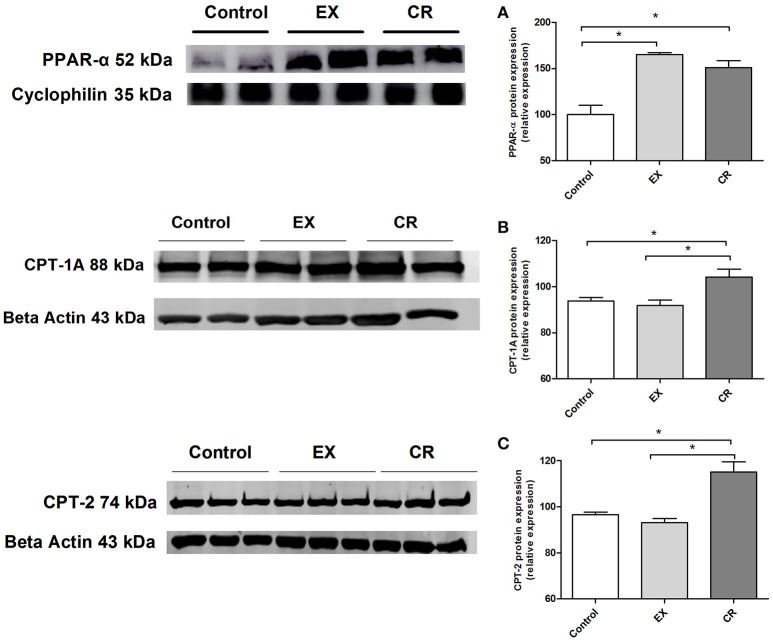
**PPAR-alpha and its target genes protein expression in the kidney**. Renal protein expression of **(A)** PPAR-alpha, **(B)** CPT-1a, and **(C)** CPT-2 in the kidneys of animals without cisplatin injection. Protein expression performed by western blotting. EX, physical exercise; CR, caloric restriction. ^*^p < 0.05. *n* = 5.

We performed western blotting to assay the expression of PPAR-α, CPT-1A and CPT-2,(two target proteins of PPAR-α activation). The protein expression of PPAR-α were increased in the CR and exercised group (Figure 8A), however only CR was capable to increase CPT-1A and CPT-2 expression (Figures 8B,C).

### An antagonist of PPAR-α blocks the protective effect of CR in urea and necrosis in cisplatin-treated mice

**Figure 9:**
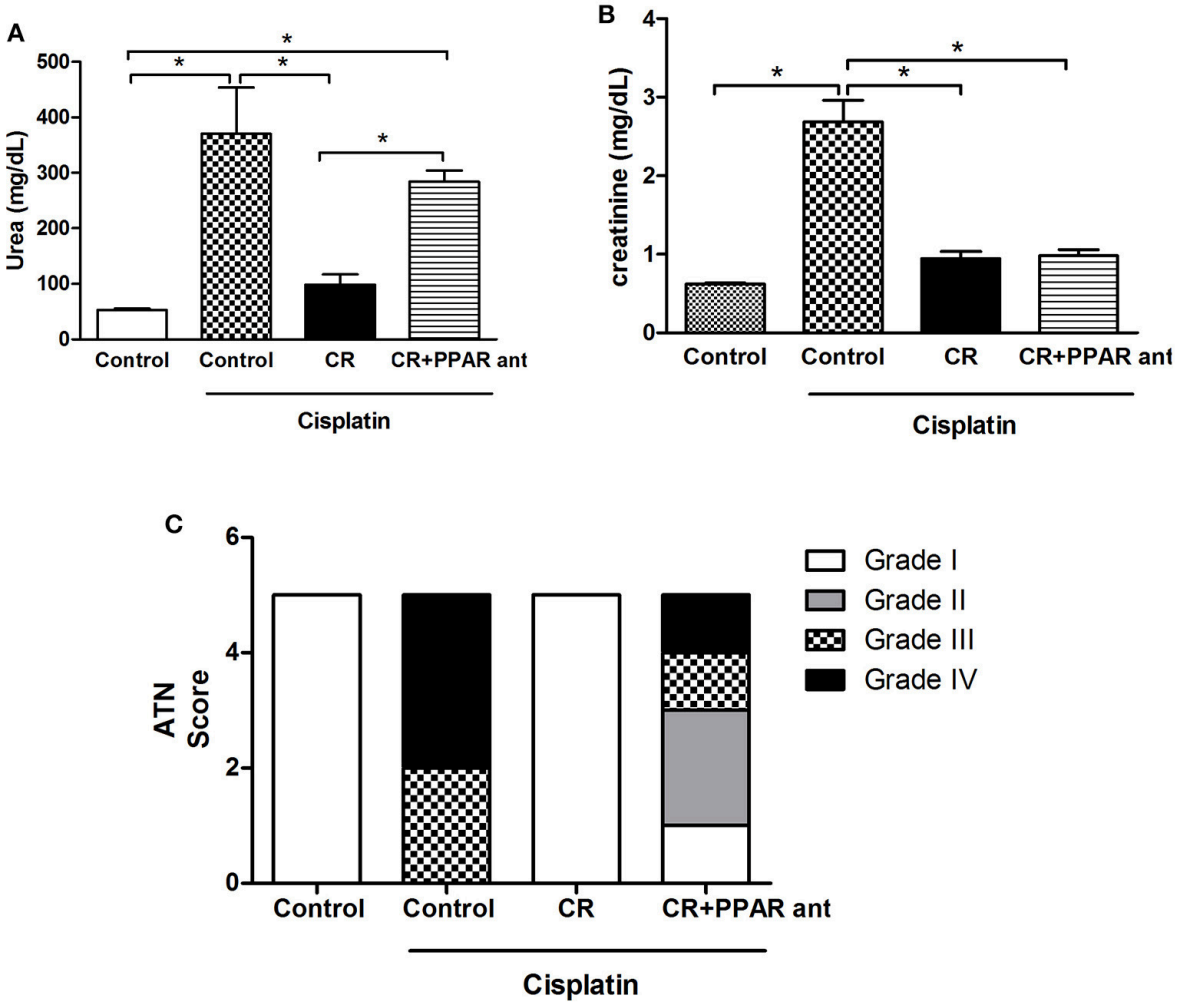
**Effect of PPAR-α antagonism on markers for renal injury in mice on CR and cisplatin treatment**. Serum levels of **(A)** Urea, and **(B)** creatinine, histological analysis of **(C)** acute tubular necrosis score. ^*^p < 0.05. *n* = 5–6.

To test if PPAR-α is involved in the CR mechanism of renal protection from cisplatin nephrotoxicity, we treated mice on CR with MK886, a PPAR-α antagonist (Kehrer et al., 2001), and compared these mice with the CIS and control groups. Mice on CR and treated with this PPAR-α antagonist and cisplatin presented similar increases in serum urea levels as animals treated only with cisplatin, when compared to the control group (Figure 9A). The same effect was not observed for the serum creatinine levels (Figure 9B). Moreover, the acute tubular necrosis score was not completely reduced in the CR group treated with MK886 (Figure 9C).

### PPAR-α antagonism blocks the beneficial effect of CR on apoptosis, Bax/Bcl2 ratio, and renal mRNA expression of TNF-α

**Figure 10:**
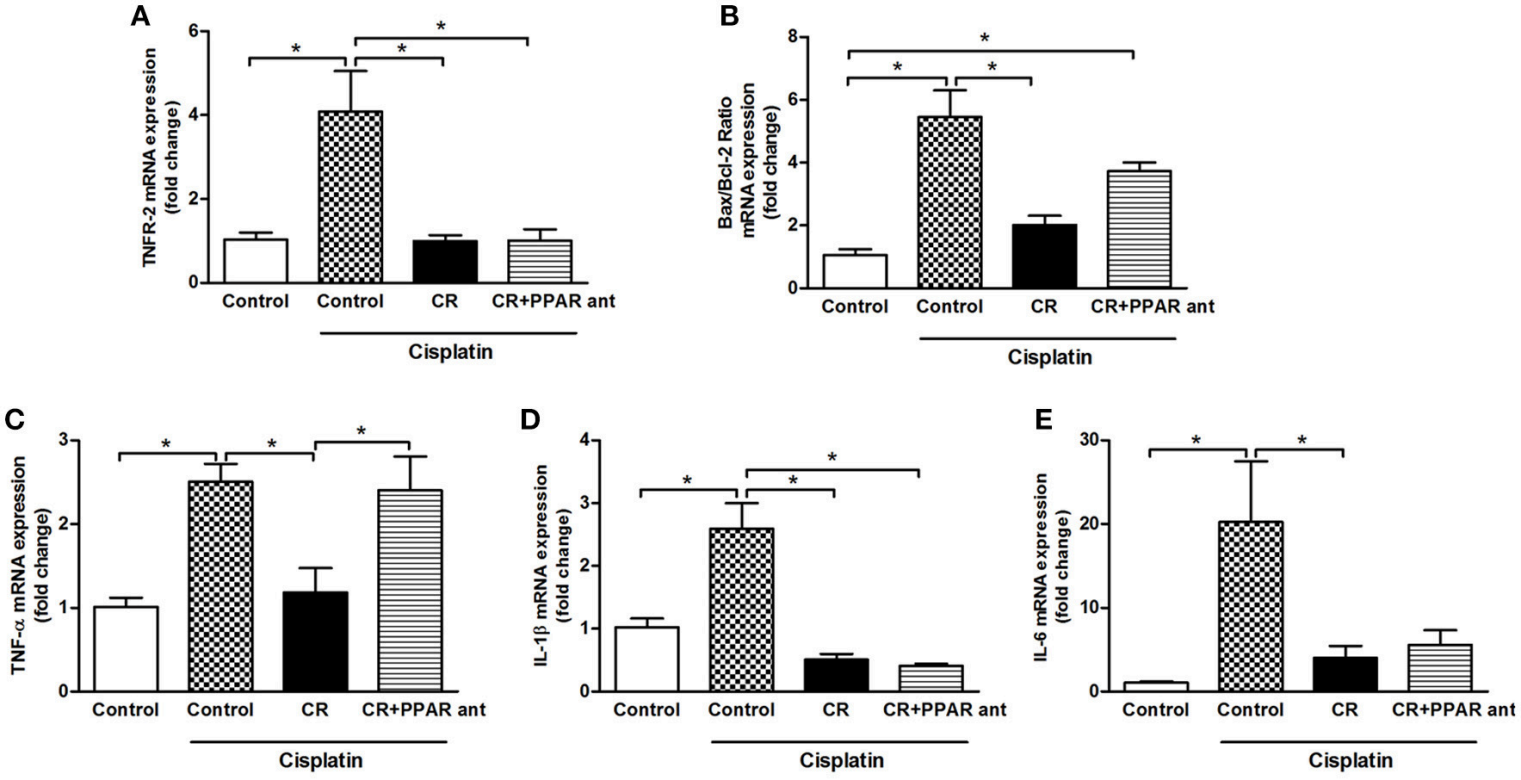
**Effect of PPAR-α antagonism on mRNA expression of apoptosis-related genes and cytokines in the kidneys of mice on CR and cisplatin treatment**. **(A)** TNFR2, **(B)** Bax/Bcl2 ratio, **(C)** TNF-alpha, **(D)** IL-1b, and **(D)** IL-6 renal mRNA expression. ^*^p < 0.05. *n* = 5–6.

We assessed the changes in renal mRNA expression in the animals on CR and treated with MK886. The cisplatin effect on TNFR2, IL-1b, and IL-6 mRNA expression was not changed by blocking PPAR-α (Figures 10A,D,E), but the beneficial effect observed on the Bax/Bcl2 ratio and TNF-α was clearly blocked (Figures 10B,C). These results can be compared to those in Figures 4, 5, where the effect of CR on the mRNA expression of these factors was markedly stronger.

## Discussion

Cisplatin is a widely used drug for various tumors. However, its deleterious side effects in the kidney limit its therapeutic use (Arany and Safirstein, 2003; Miller et al., 2010). Inflammation and apoptosis are involved in triggering the nephrotoxicity caused by cisplatin (Yao et al., 2007; Miller et al., 2010). Both physical exercise and caloric restriction (CR) are non-pharmacological interventions to mitigate the inflammatory effects of diseases such as obesity (Ahmed et al., 2009; Wasinski et al., 2013), diabetes (Solomon et al., 2008; Snel et al., 2011, 2012), cardiovascular diseases (Chandrasekar et al., 2001; Ahmet et al., 2005; Edwards et al., 2010), and kidney diseases (Friend et al., 1978; Kobayashi and Venkatachalam, 1992; Cherry et al., 1998; Mitchell et al., 2010; Miyagi et al., 2014). In this study, we show that both exercise and CR attenuate the acute renal failure induced by cisplatin, but CR has a stronger effect than exercise. For the molecular mechanism of this protection, we hypothesize that CR modulates PPAR-α activation.

The stronger effect of CR compared to exercise was observed in its effect on mitigating weight loss. Moreover, animals subjected to CR did not exhibit a sharp drop in food intake. We also observed that physical exercise and CR normalized creatinine levels, but only CR reduced the urea levels after exposure to cisplatin. Together with the evaluation of acute tubular necrosis, where we observed better protection in the group of animals subjected to CR, this finding confirms the stronger effect of CR in protecting against cisplatin renal injury compared to the physical exercise protocol.

TNF-α is the main cytokine involved in acute kidney injury induced by cisplatin (Ramesh and Reeves, 2002; Pabla and Dong, 2008). TNF-α knockout mice showed a better response when treated with cisplatin, including better renal function, reduced inflammation and lower levels of necrosis (Ramesh and Reeves, 2002). Knowing that both circulating and tissue cytokines are important in triggering acute kidney injury induced by cisplatin (Arany and Safirstein, 2003; Yao et al., 2007; Miller et al., 2010), we analyzed serum levels and tissue expression of the main cytokines and observed lower serum levels of TNF-α and IL-1β in the CR group than in exercise group. As we found no differences between physical exercise and CR for either IL-6, MCP-1, and IL-10 serum levels or mRNA expression in renal tissue, we conclude that the differences between the treatments came from another mechanism.

We did not see a significant difference in TNFR2 mRNA expression, only a downward trend in the CR group. Because small changes in mRNA expression can lead to large changes in protein levels, we analyzed TNFR2 protein expression by ELISA and found reduced expression of this receptor in the CR group. Moreover, we analyzed cleaved caspase-3 by immunohistochemistry and showed that CR provides greater protection than exercise against apoptosis. Our results corroborate with the literature, as the role of TNFR2 in the control of apoptosis and necrosis in cisplatin nephrotoxicity is well-described (Ramesh and Reeves, 2003; Pabla and Dong, 2008; Miller et al., 2010). TNFR2 has an essential role in controlling apoptosis and necrosis, and this receptor is more important than TNFR1 in mediating key events triggered by cisplatin nephrotoxicity, including necrosis, apoptosis and inflammation (Ramesh and Reeves, 2003). A previous report shows that mice with a deletion in this gene that were exposed to cisplatin exhibited improved renal function, reduced necrosis, lower levels of apoptosis and less inflammation compared to TNFR1 knockout mice and wild-type mice Apoptosis occurs by both the intrinsic pathway and extrinsic pathway in cisplatin nephrotoxicity (Pabla and Dong, 2008; Miller et al., 2010). We observed that both exercise and CR decreased the Bax/Bcl-2 gene expression ratio, suggesting a lower activation of apoptosis via the intrinsic pathway. However, physical exercise does not decrease TNFR2 expression, resulting in greater overall apoptosis and reduced protection from tubular necrosis compared to the CR group. From the data presented here, we showed that the main difference between the two interventions is that CR can modulate TNFR2, resulting in a better response to cisplatin-induced acute renal injury.

Knowing that both exercise and CR modulate PPAR-α (Iemitsu et al., 2002; Corton et al., 2004; Zhang et al., 2011; Lempiäinen et al., 2013), we analyzed the expression of PPAR-α and its target genes. PPAR-α is well-known as a nuclear sensor controlling lipid metabolism mainly in the liver and kidneys (Braissant et al., 1996). Deletion of this gene causes deterioration in models of acute renal failure induced by cisplatin or ischaemia and reperfusion. Treatment with activators of PPAR-α, such as fibrates, can mitigate the deleterious effects of cisplatin and ischaemia and reperfusion (Li et al., 2004a,b, 2005). Animals subjected to CR and exercise showed increased PPAR-α protein expression, in the other hand only CR were capable to increase mRNA and protein levels of PPAR-α target genes. Like other nuclear hormone receptors, PPAR acts as a ligand-activated transcription factor. PPAR-α, when activated after binding with specific ligand, interacts with RXR and regulates the expression of target genes (Cuzzocrea, 2006). Furthermore, a PPAR-α antagonist partially removed the benefits caused by CR. Urea levels and tubular necrosis scores were not reduced by CR when combined with MK886 injection. Moreover, TNF-α mRNA levels and Bax/Bcl-2 ratios were not diminished. Here, we demonstrated that PPAR-α participates in the mechanism by which CR protects against cisplatin-induced acute kidney injury, connecting the effect of CR in PPAR-α activation and its mechanism as a protector to mitigate cisplatin-induced renal injury.

The data collected here show for the first time that both CR and physical exercise attenuate the nephrotoxicity caused by cisplatin injection, but CR shows a better response by modulating PPAR-α activation. Moreover, we showed that part of the CR benefit depends on PPAR-α. Further studies are necessary, but we suggest that CR might be a good tool to prevent the nephrotoxicity induced by cisplatin during chemotherapy treatment.

## Ethics statement

CEUA/UNIFESP project number 155783.

## Author contributions

Design of work: GE, RA, MB, and NC. Performed experiments: GE, FW, RB, RF, MH, FC, DM, and DdA. Data Analysis: GE, FW, RB, RF, FC, DM, CB, NC, and RA. Wrote paper: GE, CB, MB, and RA.

### Conflict of interest statement

The authors declare that the research was conducted in the absence of any commercial or financial relationships that could be construed as a potential conflict of interest. The reviewer RH and handling Editor declared their shared affiliation, and the handling Editor states that the process nevertheless met the standards of a fair and objective review.
